# Analysis of the efficacy of subclinical doses of esketamine in combination with propofol in non-intubated general anesthesia procedures - a systematic review and meta-analysis

**DOI:** 10.1186/s12871-023-02135-8

**Published:** 2023-07-21

**Authors:** Haoming Chen, Xizhi Ding, Guilin Xiang, Liu Xu, Qian Liu, Qiang Fu, Peng Li

**Affiliations:** 1 Department of Anesthesiology, Affiliated Hospital of Southwest Medical University, Luzhou, China; 2 Department of Anesthesiology, Sichuan Provincial People’s Hospital, Sichuan Academy of Medical Sciences, University of Electronic Science and Technology of China, Chengdu, China; 3 Chengdu Medical College, Chengdu, China; 4 Wenjiang Hospital of Sichuan Provincial People’s Hospital, Chengdu, China; 5 Department of Anesthesiology, The Third People’s Hospital of Chengdu, Chengdu, China; 6 Department of Anesthesiology, The First People’s Hospital of Guangyuan, Guangyuan, China

**Keywords:** Propofol, Esketamine, Non-intubated general anesthesia, Hemodynamics, Adverse reactions

## Abstract

**Background:**

The number of non-intubated general anesthesia outside the operating room is growing as the increasing demand for comfort treatment. Non-intubated general anesthesia outside the operating room requires rapid onset of anesthesia, smoothness, quick recovery, and few postoperative complications. Traditional anesthetic regimens (propofol alone or propofol and opioids/dezocine/midazolam, etc.) have severe respiratory and circulatory depression and many systemic adverse effects. In this paper, we compare the effectiveness and safety of propofol and subclinical doses of esketamine with other traditional regimens applied to non-intubated general anesthesia through a systematic review and meta-analysis.

**Methods:**

We searched PubMed, Embase, Cochrane Library, Web of Science, CNKI, Wanfang, VIP, and Sinomed databases for the period from January 2000 to October 2022. We rigorously screened the literature according to predefined inclusion and exclusion criteria, while risk assessment of the studies was performed using The Cochrane Collaboration’s tool, and statistical analysis of the data was performed using RevMan 5.4 software. The main outcome indicators we evaluated were the various hemodynamic parameters and incidence of various adverse effects between the experimental and control groups after induction of anesthesia.

**Results:**

After a rigorous screening process, a total of 14 papers were included in the final meta-analysis. After risk bias assessment, three of the papers were judged as low risk and the others were judged as having moderate to high risk. Forest plots were drawn for a total of 16 indicators. Meta-analysis showed statistically significant differences in HR’ WMD 3.27 (0.66, 5.87), MAP’ WMD 9.68 (6.13, 13.24), SBP’ WMD 5.42 (2.11, 8.73), DBP’ WMD 4.02 (1.15, 6.88), propofol dose’ SMD -1.39 (-2.45, -0.33), hypotension’ RR 0.30 (0.20, 0.45), bradycardia’ RR 0.33 (0.14, 0.77), hypoxemia or apnea’ RR 0.45 (0.23, 0.89), injection pain’ RR 0.28 (0.13, 0.60), intraoperative choking’ RR 0.62 (0.50, 0.77), intraoperative body movements’ RR 0.48 (0.29, 0.81) and overall incidence of adverse reactions’ RR 0.52 (0.39, 0.70).The indicators that were not statistically different were time to wake up’ WMD − 0.55 (-1.29, 0.19), nausea and vomiting 0.84’ RR (0.43, 1.67), headache and dizziness’ RR 1.57 (0.98, 2.50) and neuropsychiatric reaction’ RR 1.05 (0.28, 3.93). The funnel plot showed that the vast majority of studies fell within the funnel interval, but the symmetry was relatively poor.

**Conclusion:**

In non-intubated general anesthesia, the combination of subclinical doses of esketamine and propofol did reduce circulatory and respiratory depression, injection pain, and other adverse effects, while the incidence of esketamine’s own side effects such as neuropsychiatric reactions did not increase, and the combination of the two did not cause the occurrence of new and more serious adverse reactions, and the combination of the two was safe and effective.

**Trial registration:**

PROSPREO registration number: CRD 42022368966.

## Introduction

With the increasing popularity of comfort medicine, many people are choosing general anesthesia when they receive treatment, such as painless gastroenteroscopy, painless bronchoscopy, painless abortion, etc [1]. Many procedures that could previously be performed under local anesthesia are now being performed under general anesthesia for a better medical experience. As a result, the proportion of non-intubated general anesthesia outside the operating room is increasing dramatically. The risks of non-intubated general anesthesia outside the operating room cannot be underestimated due to the limitations of the operating site, equipment conditions, and anesthesia procedures. Non-intubated general anesthesia outside the operating room requires rapid onset of anesthesia, smoothness, rapid recovery, and few postoperative complications, which requires a very safe and effective anesthetic drug regimen.

Propofol is now basically one of the essential drugs for non-intubated general anesthesia outside the operating room, with rapid onset of action, smooth induction, rapid metabolism, and no side effects such as involuntary muscle tremors, etc [2]. Although propofol has many advantages that cannot be compared with other intravenous anesthetics, it still has some adverse effects that cannot be ignored, such as respiratory and circulatory depression, injection pain, etc [3]. Therefore, this creates the need to use other drugs in combination with propofol to minimize these side effects of propofol. But there is no consensus about which drug and propofol is the best pairing options [4]. Currently, the commonly used drugs in clinical practice are opioids (fentanyl, sufentanil, etc.), benzodiazepines (midazolam, etc.), and lidocaine, but because opioids and benzodiazepines themselves have strong side effects such as respiratory and circulatory depression, combining with propofol greatly increases the difficulty of respiratory and circulatory management, we considered whether there are more proper drug would possess better efficacy when paired with propofol [5–8]. Given the unique pharmacological characteristics of esketamine, it came into our consideration.

Esketamine, as the right-hand molecular structure of ketamine, acts like ketamine mainly by antagonizing the N-methyl-D-aspartate receptor [9]. However, compared to ketamine, esketamine has about twice the analgesic potency, so it can achieve the same effect as ketamine through a smaller dose, which can greatly reduce the occurrence of side effects such as neuropsychiatric reactions and secretion production during the awakening period [10]. Because of its sympathomimetic effect, we considered esketamine in combination with propofol, which can buffer the violent fluctuations of the circulation [11]. At the same time, esketamine has less effect on respiration and has analgesic effect, which can effectively compensate for the respiratory depression and injection pain caused by propofol [12]. Based on their pharmacological characteristics, we conclude that the combination of the two is very complementary in non-intubated general anesthesia procedures outside the operating room. The current clinical dose of esketamine alone for induction of anesthesia is usually 0.5-1 mg/kg, but the incidence of neuropsychiatric and other adverse effects caused by esketamine in this dose range is still high, while some studies have shown that subclinical doses (less than 0.5 mg/kg) of esketamine can produce analgesia with relatively few neuropsychiatric and other side effects [10, 13]. Many randomized controlled trials have been conducted to examine the efficacy of subclinical doses of esketamine and propofol together, and our study compares the efficacy and safety of propofol and subclinical doses of esketamine with other conventional regimens (propofol alone or propofol and opioids/dezocine/midazolam, etc.) applied to non-intubated general anesthesia through a systematic review and meta-analysis.

## Methods

### Search strategy

A computer-based search of databases including PubMed, Embase, Cochrane Library, Web of Science, CNKI, Wanfang, VIP, Sinomed. The search strategy was as follows:(“Propofol”) AND (“Esketamine”). The date parameter for the search was set from January 2000 to October 2022.

### Inclusion and exclusion criteria

The inclusion criteria are as follows: (a) randomized controlled trials on humans, (b) non-intubated general anesthesia without any muscle relaxants, (c) only two anesthetics, propofol and esketamine, were used in the experimental group (d) subclinical doses (0.5 mg/kg less) of esketamine, and (e) adults over 18 years old.

The exclusion criteria are as follows: (a) data missing and (b) repeatedly published articles.

### Quality assessment

All included articles were assessed independently by the review authors using The Cochrane Collaboration’s tool for assessing risk of bias. Disagreements and discrepancies were solved through a consensus discussion with correspondence author. The risk of bias was graded as high, uncertain or low according to the following seven critical domains with respect to The Cochrane Collaboration’s tool for assessing risk of bias: random sequence generation, allocation concealment, blinding of participants and personnel, blinding of outcome assessment, incomplete outcome data, selective reporting and other bias. The Cochrane Collaboration’s tool for assessing risk of bias proposes an approach for summarizing the risk of bias according to the seven domains, which was used judiciously. A study was judged to have low risk of bias when there was low risk of bias for all key domains and plausible bias was unlikely to seriously alter the results. Unclear risk of bias for one or more key domains resulted in an overall unclear risk of bias for the study, while high risk of bias for any domain resulted in an overall high risk of bias for the study.

### Data extraction

To clarify the efficacy and incidence of adverse effects of different drug regimens in non-intubated anesthesia, we extracted basic information for each article (including author and year of publication), number of subjects and age groups, trial design protocol (dosing regimen for experimental and control groups), and results on the incidence of various adverse effects (including, but not limited to, e.g., hemodynamic parameters, respiratory depression, injection pain, etc.)

### Statistical analysis

Statistical analysis of the data was performed using RevMan 5.4 (Cochrane Collaboration). According to the heterogeneity test results, the effect model is determined. I2 ≥ 50% indicates greater heterogeneity, and the random effect model is selected; I2 < 50% indicates that the heterogeneity is within the acceptable range, and the fixed effect model is selected. When P < 0.05, it was considered that there were significant differences in the changes of each outcome indices. The weighted mean difference (WMD) is used to represent the results of measurements using the same unit of measurement; otherwise, the standardized mean difference (SMD) is used to represent the results. All results were expressed with 95% confidence intervals (CI). Sensitivity analysis was used to assess the reliability and stability of the results. Funnel plots were drawn, and the publication bias was evaluated by the symmetry of the funnel plot and concentration of literature to the midline.

## Results

### Literature search results and profile analysis

**Fig. 1:**
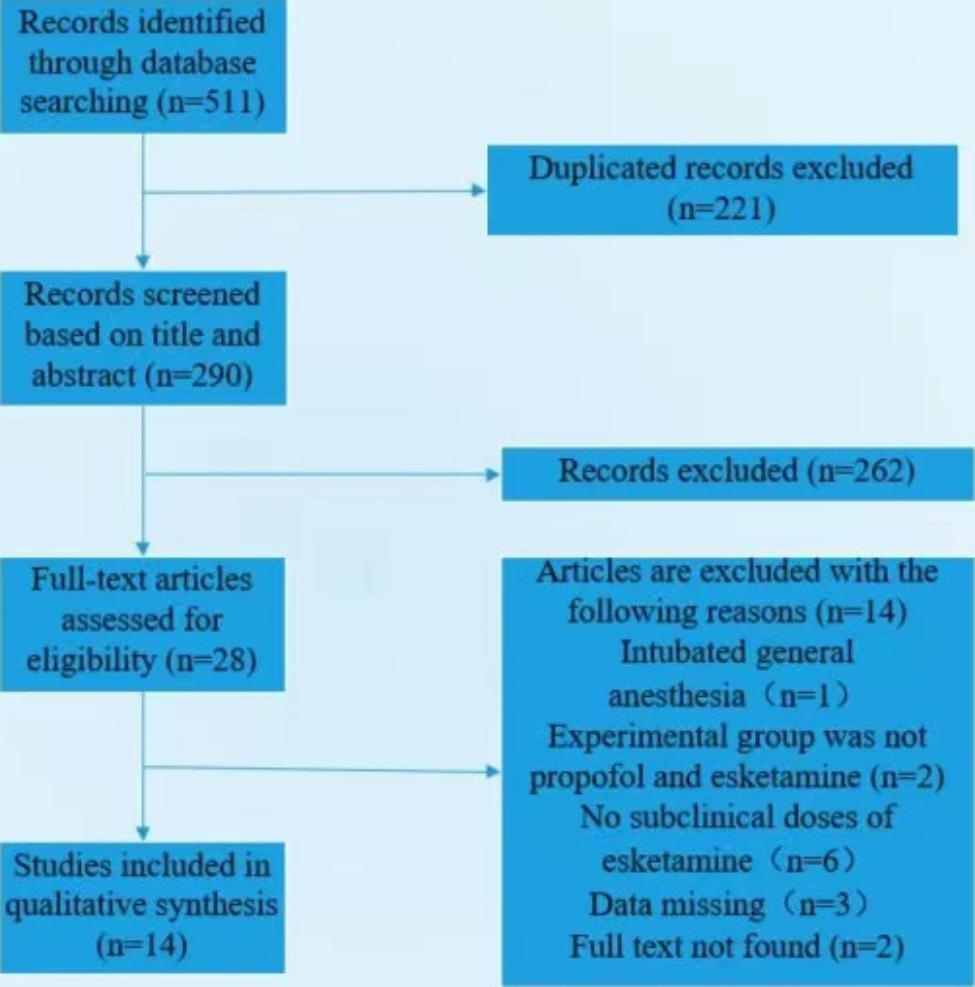
Flowchart demonstrating the process of inclusion and exclusion of articles

Pubmed searched 35 articles, Embase searched 135 articles, Cochrane Library searched 82 articles, web of science searched 32 articles, CNKI searched 74 articles, Wanfang searched 60 articles, VIP searched 37 articles, and Sinomed searched 56 articles, and a total of 14 articles were included in this review after being screened strictly according to the inclusion and exclusion criteria, and the screening process of these articles is shown in Fig. 1. The grouping of each study with outcome indicators and other information are summarized in Table 1.

**Table 1:** Characteristics of included RCTs

Examination performed	Participants	Sequence of propofol and esketamine	Experimental groups	Control groups	Outcome indicators
Painless abortion [24]	178 woman patients aged 18–45 years	Esketamine injected first	EL group (propofol + 0.2 mg/kg esketamine, n = 45), EM group (propofol + 0.25 mg/kg esketamine, n = 44), EH group (propofol + 0.3 mg/kg esketamine, n = 44)	GF group (propofol + 1ug/kg fentanyl, n = 45)	HR, MAP, hypotension, hypoxemia or apnea, nausea and vomiting, intraoperative body movements, neuropsychiatric symptoms, total incidence of adverse reactions
ERCP [25]	162 patients aged 18 years and above	Propofol injected first	Propofol + 0.15 mg/kg esketamine, n = 83	Propofol + 2ug/kg alfentanil,n = 79	Propofol dosage, hypotension, bradycardia, hypoxemia or apena, total incidence of adverse reactions
Painless gastroscopy [26]	105 patients aged 18–80 years	Esketamine injected first	Group A (propofol + 0.3 mg/kg esketamine, n = 35),	Group C (propofol + saline, n = 35)	HR, MAP, propofol dosage, wake-up time, postoperative VAS score, injection pain, hypotension, bradycardia, hypoxemia or apnea, nausea and vomiting, agitation during the awakening period, total incidence of adverse reactions
Painless gastroscopy [4]	83 patients aged 18–64 years	Esketamine injected first	Propofol + 0.3 mg/kg esketamine, n = 42	Propofol + 0.05 mg/kg dezocine, n = 41	HR, MAP, propofol dosage, injection pain,hypotension, bradycardia, hypoxemia or apnea, nausea and vomiting, agitation during the awakening period, total incidence of adverse reactions
Painless gastroenteroscopy [27]	260 patients aged 18–60 years	Esketamine injected first	PK1 group (propofol + 0.05 mg/kg esketamine, n = 65), PK2 group (propofol + 0.1 mg/kg esketamine, n = 65), PK3 group (propofol + 0.2 mg/kg esketamine, n = 65)	Propofol + saline, n = 65	HR, SBP, DBP, propofol dosage, injection pain, hypotension, hypoxemia or apnea, intraoperative cough, intraoperative body movements, headache or dizziness, total incidence of adverse reactions
Painless gastroenteroscopy [28]	102 patients aged 20–69 years	Esketamine injected first	Propofol + 0.25 mg/kg esketamine, n = 51	Only propofol, n = 51	HR, SBP, DBP, wake-up time, hypotension, hypoxemia or apnea, intraoperative cough, headache or dizziness, neuropsychiatric symptoms, total incidence of adverse reactions
Painless abortion [29]	159 woman patients aged 18 years and above	Esketamine injected first	Propofol + 0.25 mg/kg esketamine, n = 80	Propofol + 0.1ug/kg sufentanil, n = 79	propofol dosage, wake-up time, postoperative VAS score
minimally invasive surgery of benign breast tumors [30]	60 woman patients aged 20–60 years	Unknown	Propofol + 0.4 mg/kg esketamine, n = 30	Propofol + 2ug/kg remifentanil, n = 30	wake-up time, bradycardia, intraoperative cough, nausea and vomiting, headache or dizziness, total incidence of adverse reactions
Painless gastroenteroscopy [31]	60 patients aged 18–65 years	Esketamine injected first	Propofol + 0.2 mg/kg esketamine, n = 30	Propofol + saline, n = 30	HR, MAP, propofol dosage, wake-up time, hypotension, bradycardia, hypoxemia or apnea, intraoperative body movements, total incidence of adverse reactions
Painless gastroenteroscopy [32]	102 patients aged 18 years and above	Esketamine injected first	Group A (propofol + 0.2 mg/kg esketamine, n = 34),	Group C (propofol + 0.5ug/kg remifentanil, n = 34)	HR, SBP, DBP, hypoxemia or apnea, nausea and vomiting, intraoperative body movements, total incidence of adverse reactions
Painless gastroenteroscopy [33]	120 patients aged 65–85 years	Esketamine injected first	Group C (propofol + 0.25 mg/kg esketamine, n = 40)	Group A (propofol + 0.08 mg/kg dezocine, n = 40), Group B (propofol + 0.08ug/kg sufentanil, n = 40)	HR, MAP, hypotension, hypoxemia or apnea, intraoperative body movements, total incidence of adverse reactions
Painless bronchoscopy [34]	94 patients aged 20–75 years	Esketamine injected first	Propofol + 0.1 mg/kg esketamine, n = 47	Propofol + 0.4ug/kg sufentanil, n = 47	HR, SBP, DBP, hypoxemia or apnea, intraoperative cough, nausea and vomiting, total incidence of adverse reactions
Painless gastroenteroscopy [35]	100 patients aged 18–74 years	Esketamine injected first	Propofol + 0.25 mg/kg esketamine, n = 50	Only propofol, n = 50	HR, SBP, DBP, wake-up time, postoperative VAS score, injection pain, hypoxemia or apnea, intraoperative cough, intraoperative body movements, headache or dizziness, total incidence of adverse reactions
Painless abortion [36]	90 woman patients aged 20–35 years	Esketamine injected first	Group one (propofol + 0.25 mg/kg esketamine, n = 30)	Group two (propofol + 0.1 mg/kg dezocine, n = 30), Group three (only propofol, n = 30)	HR, MAP, wake-up time, injection pain, nausea and vomiting, intraoperative body movements, headache or dizziness, neuropsychiatric symptoms, total incidence of adverse reactions

HR: heart rate, SBP: systolic blood pressure, DBP: diastolic blood pressure, MAP: mean arterial pressure, ERCP: endoscopic retrograde cholangiopancreatography

### Bias risk assessment of included literatures

**Fig. 2:**
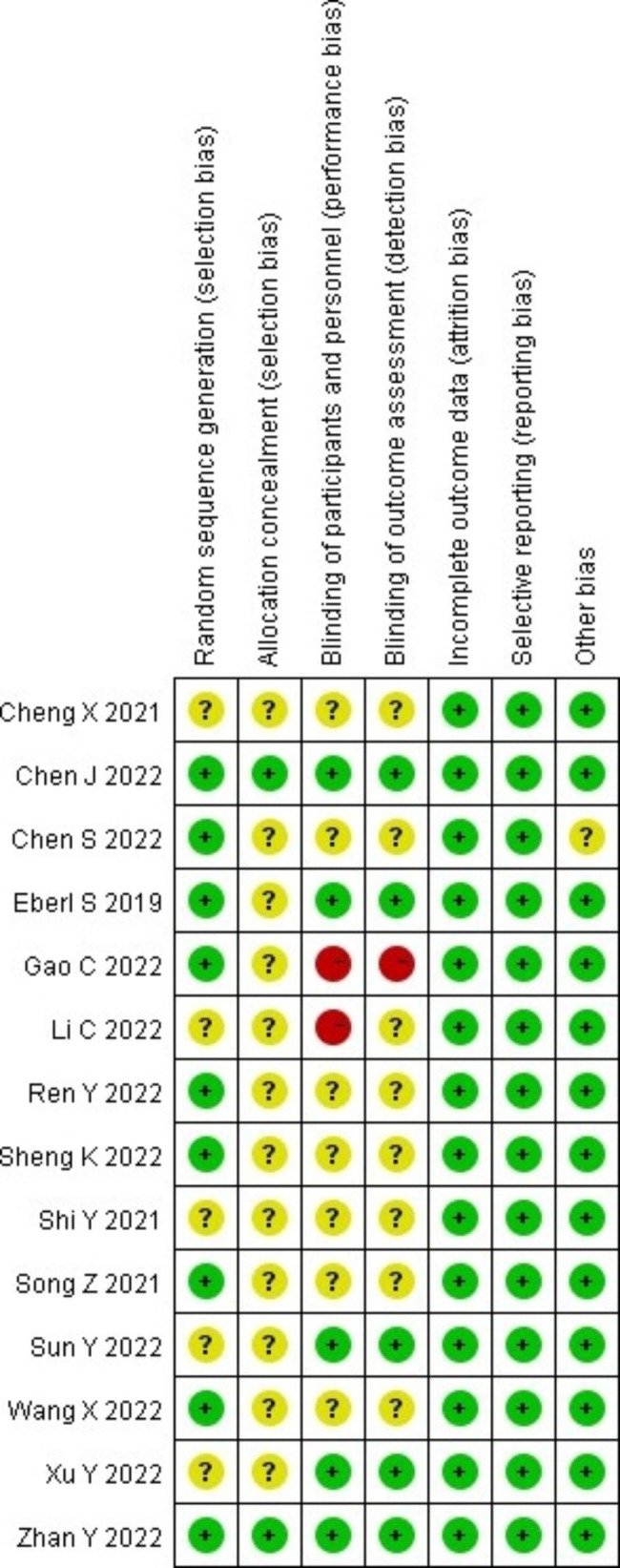
Risk of bias summary of the included literatures

**Fig. 3:**
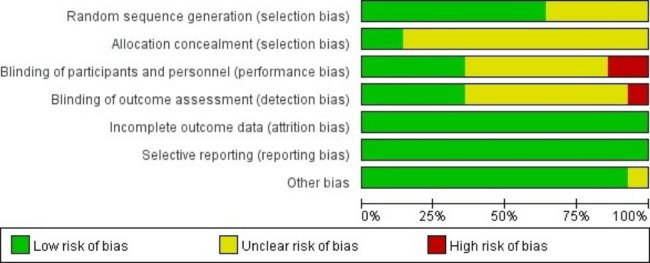
Risk of bias graph

The bias risk assessment tool recommended by the Cochrane Handbook for Systematic Reviews of Interventions was used to evaluate the quality of the included literatures, and the results are shown in Figs. 2 and 3. Articles Chen J 2022, Eberl S 2019 and Zhan Y 2022 were rated as having a low risk, and the remaining studies were rated as having a moderate to high risk.

### Pooled analysis

**Fig. 4:**
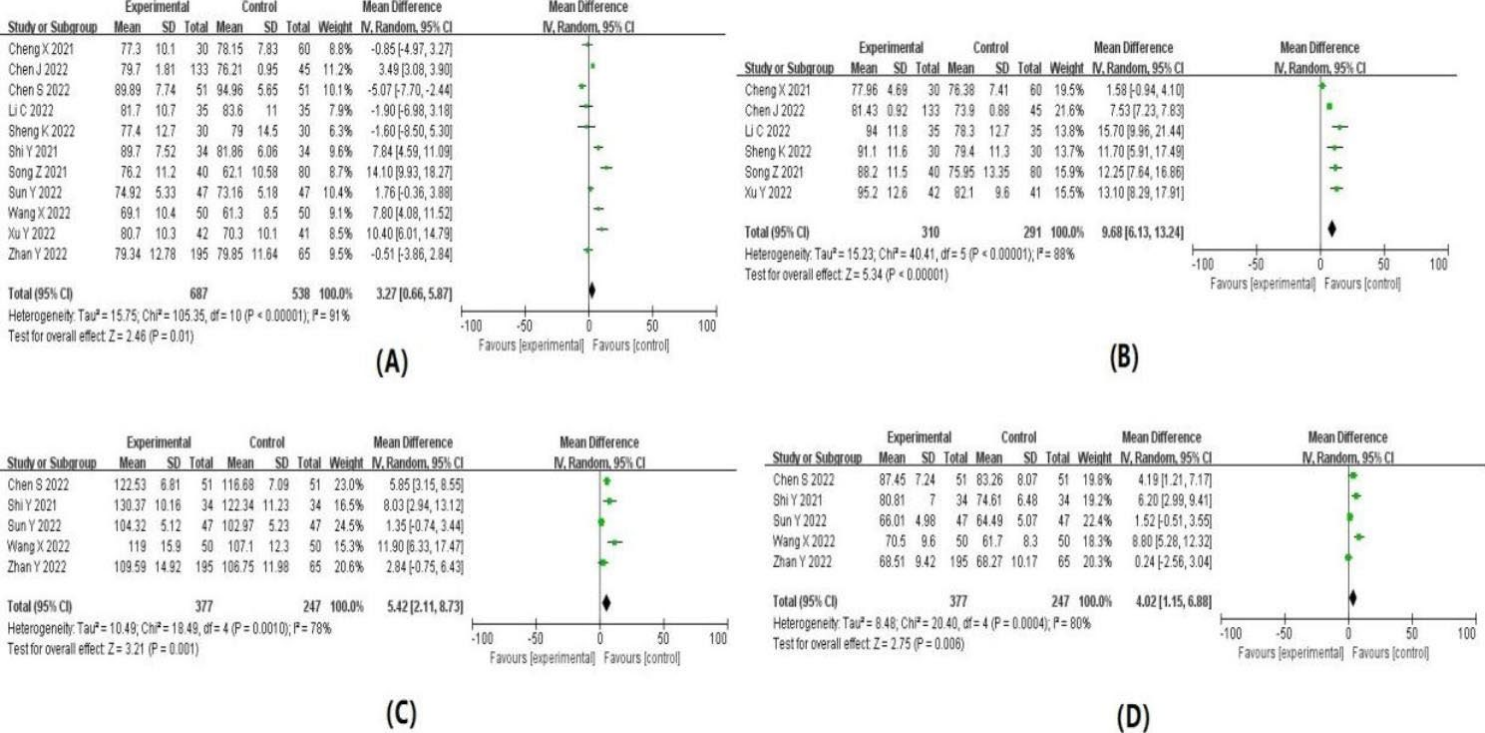
Forest plot of hemodynamic indices after induction of anesthesia. (**A**) heart rates; (**B**) mean arterial pressure; (**C**) systolic blood pressure; (**D**) diastolic blood pressure

**Fig. 5:**
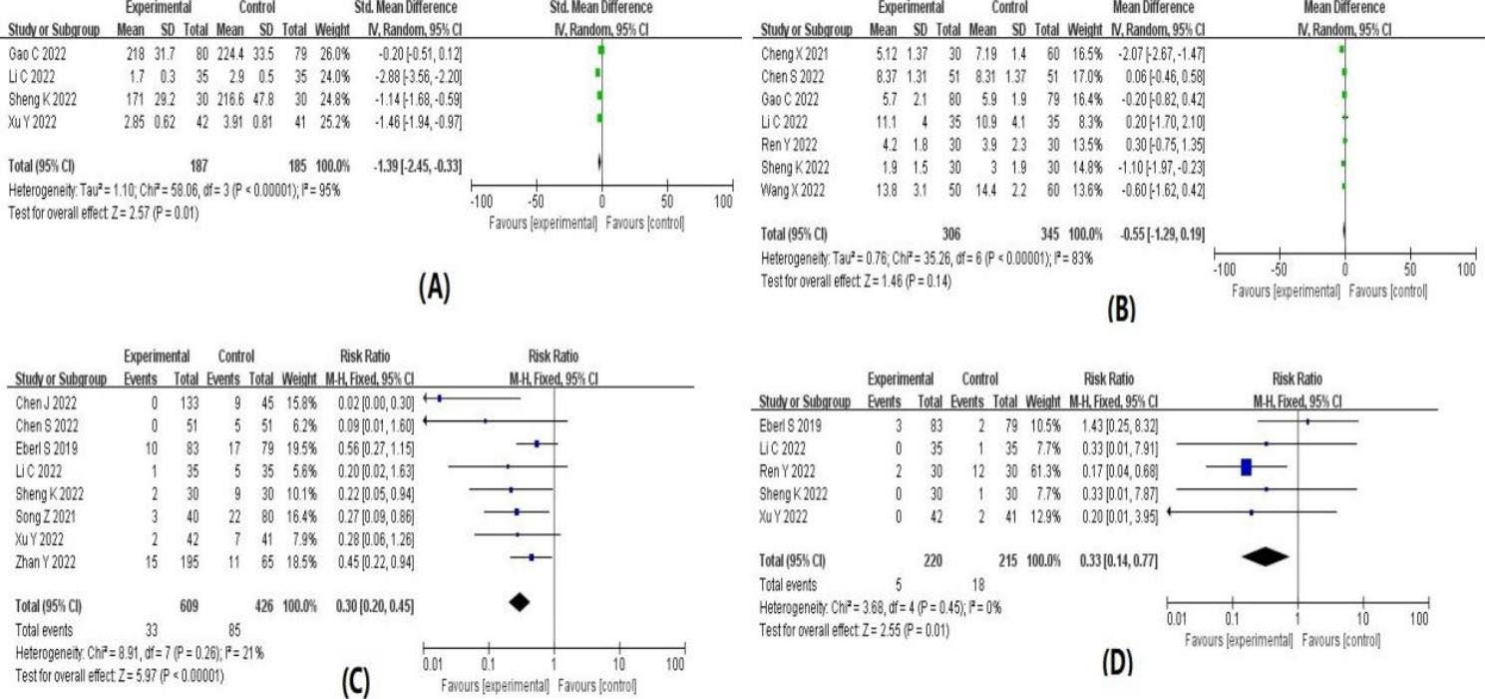
Forest plot of (**A**) propofol dose; (**B**) awakening time; (**C**) hypotension; (**D**) bradycardia

**Fig. 6:**
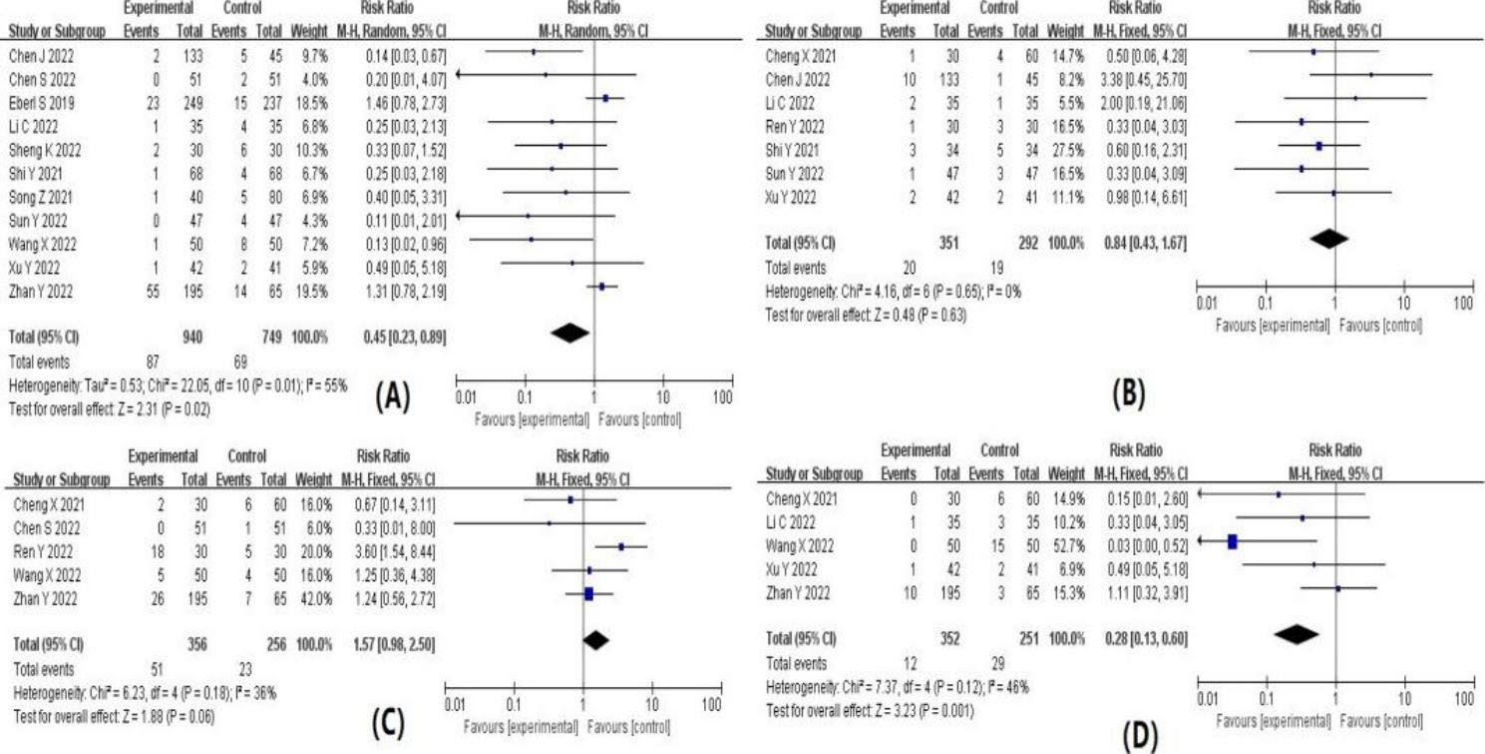
(**A**) hypoxemia or apnea; (**B**) nausea and vomiting; (**C**) headache and dizziness; (**D**) injection pain

**Fig. 7:**
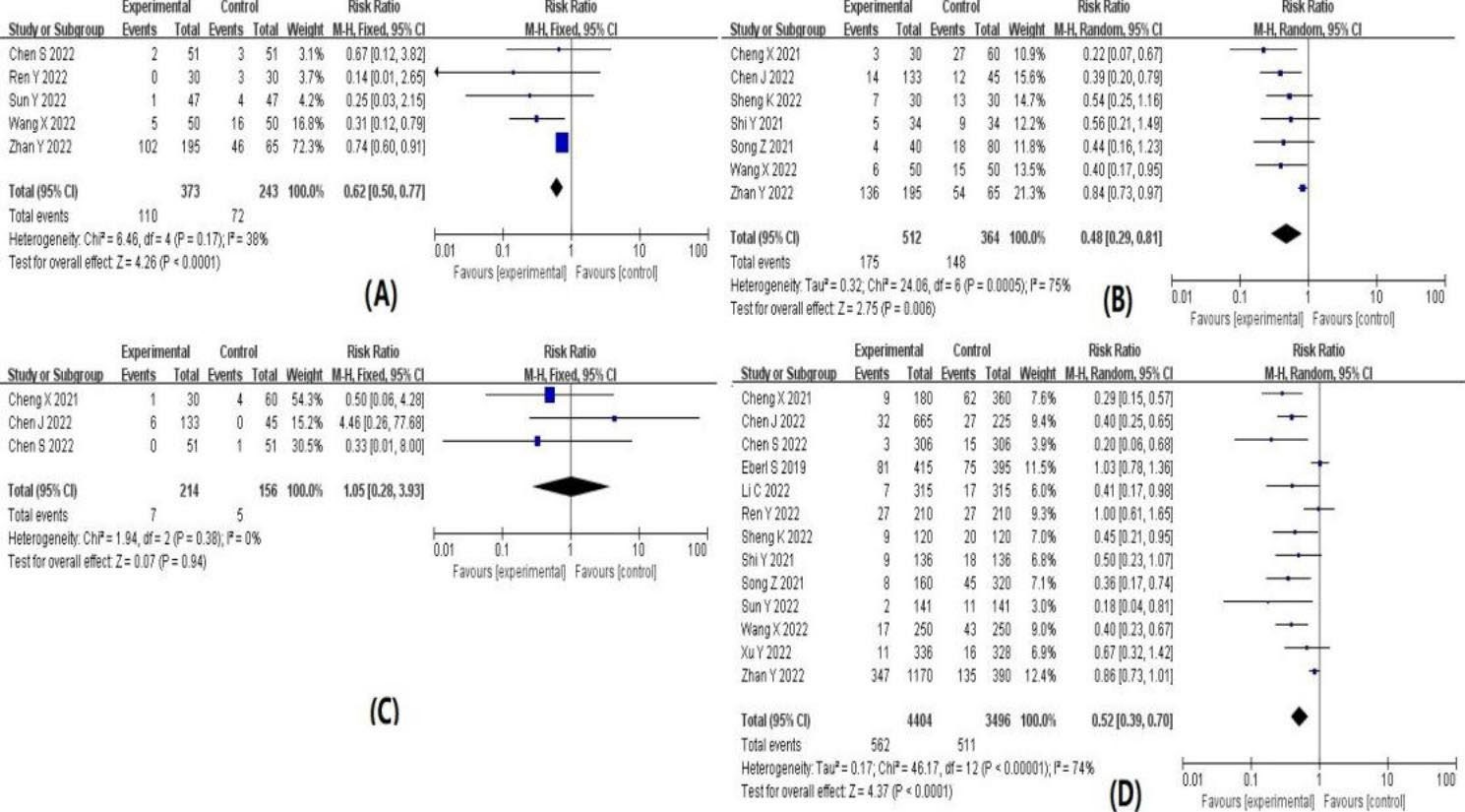
(**A**) intraoperative choking; (**B**) intraoperative body movements; (**C**) neuropsychiatric reactions; (**D**) total adverse effects

The results of the meta-analysis are summarized in Table 2. Forest plots for each indicator are shown in detail in Figs. 4, 5, 6 and 7. The indicators that showed statistical differences were HR’ WMD 3.27 (0.66, 5.87), MAP’ WMD 9.68 (6.13, 13.24), SBP’ WMD 5.42 (2.11, 8.73), DBP’ WMD 4.02 (1.15, 6.88), propofol dose’ SMD -1.39 (-2.45, -0.33), hypotension’ RR 0.30 (0.20, 0.45), bradycardia’ RR 0.33 (0.14, 0.77), hypoxemia or apnea’ RR 0.45 (0.23, 0.89), injection pain’ RR 0.28 (0.13, 0.60), intraoperative choking’ RR 0.62 (0.50, 0.77), intraoperative body movements’ RR 0.48 (0.29, 0.81) and overall incidence of adverse reactions’ RR 0.52 (0.39, 0.70), while the indicators that did not show statistical differences were time to wake up’ WMD − 0.55 (-1.29, 0.19), nausea and vomiting 0.84’ RR (0.43, 1.67), headache and dizziness’ RR 1.57 (0.98, 2.50) and neuropsychiatric reaction’ RR 1.05 (0.28, 3.93). Meta-analysis’ results confirmed that subclinical doses of esketamine and propofol in combination slowed the dramatic fluctuations of hemodynamic parameters (HR, MAP, SBP, DBP), reduced the dose of propofol, and reduced the incidence of hypotension, bradycardia, hypoxemia and apnea, injection pain, intraoperative choking, intraoperative body movements, and overall adverse effects compared with propofol and other drugs.

**Table 2:** Results of meta-analysis

Outcome indicators	Included study	Heterogeneity test results		Effect model	Results of meat analysis	
		I^2^ value(%)	P value		WMD, SMD or RR(95%CI)	P value
HR	11	91	P < 0.00001	Random	3.27(0.66,5.87)	0.01
MAP	6	88	P < 0.00001	Random	9.68(6.13,13.24)	P < 0.00001
SBP	5	78	P = 0.0010	Random	5.42(2.11,8.73)	P = 0.001
DBP	5	80	P = 0.0004	Random	4.02(1.15,6.88)	P = 0.006
propofol dosage	4	95	P < 0.00001	Random	-1.39(-2.45,-0.33)	P = 0.01
wake-up time	7	83	P < 0.00001	Random	-0.55(-1.29,0.19)	P = 0.14
hypotension	8	21	P = 0.26	Fixed	0.30(0.20,0.45)	P < 0.00001
bradycardia	5	0	P = 0.45	Fixed	0.33(0.14,0.77)	P = 0.01
hypoxemia and apnea	11	55	P = 0.01	Random	0.45(0.23,0.89)	P = 0.02
nausea and vomiting	7	0	P = 0.65	Fixed	0.84(0.43,1.67)	P = 0.63
headache and dizziness	5	36	P = 0.18	Fixed	1.57(0.98,2.50)	P = 0.06
injection pain	5	46	P = 0.12	Fixed	0.28(0.13,0.60)	P = 0.001
intraoperative cough	5	38	P = 0.17	Fixed	0.62(0.50,0.77)	P < 0.0001
intraoperative body movements	7	75	P = 0.0005	Random	0.48(0.29,0.81)	P = 0.006
neuropsychiatric symptoms	3	0	P = 0.38	Fixed	1.05(0.28,3.93)	P = 0.94
total incidence of adverse reactions	13	74	P < 0.00001	Random	0.52(0.39,0.70)	P < 0.0001

### Publication bias analysis

**Fig. 8:**
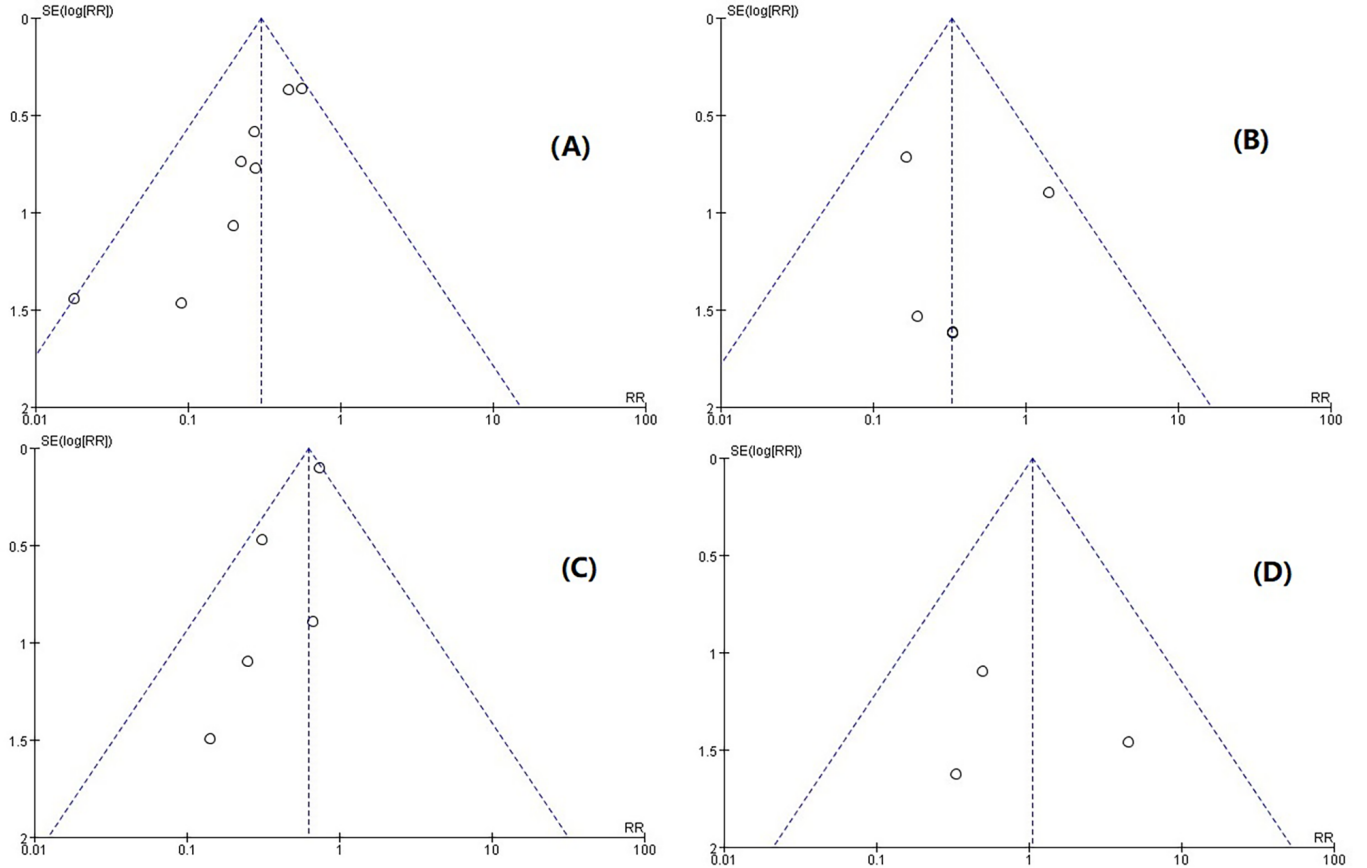
Funnel plots for evaluation indicators (**A**) hypotension; (**B**) bradycardia; (**C**) intraoperative choking; (**D**) neuropsychiatric reaction

We will analyze publication bias by plotting funnel plots using fixed-effect models with statistically significant meta-analysis’s results, including hypotension, bradycardia, intraoperative cough, and neuropsychiatric response, and the results are shown in Fig. 8. The data in all four plots are relatively concentrated. However, the symmetry of the two funnel plots, hypotension and intraoperative choking, was not particularly good, indicating a possible publication bias.

## Discussion

Based on the results of the meta-analysis, we can conclude that the combination of propofol and subclinical doses of esketamine in non-intubated general anesthesia does result in smoother hemodynamic (heart rate and blood pressure) fluctuations and a reduced incidence of hypotension and bradycardia in patients, as we previously hypothesized. Propofol has a significant depressant effect on the cardiovascular system, resulting in a decrease in cardiac output, cardiac index, per-beat index and total peripheral resistance during induction of anesthesia, due to the dual effect of peripheral vasodilation and direct cardiac depression [14]. The sympathomimetic effect of esketamine excites the sympathetic nerve center and increases the release of endogenous catecholamines, and also inhibits the reuptake of norepinephrine, which can reduce the inhibition of the cardiovascular system by propofol, an advantage that other drugs do not have in combination with propofol [15]. However, it should be noted that the sympathomimetic effect of ketamine is positive only in patients with normal sympathetic nervous system activity; otherwise, a flip-flop effect may occur [12]. Respiratory depression and apnea induced by clinical doses of propofol anesthesia is another major problem that cannot be ignored [16]. The combination of esketamine and propofol will definitely reduce the amount of propofol used, which will help to mitigate this side effect. At the same time, compared with opioids, the induction dose of clinical ketamine intravenously has only mild respiratory depression, but it can be recovered quickly, and the subclinical dose of esketamine may not worry about this problem, so the combination of the two drugs can significantly reduce the occurrence of respiratory depression and apnea compared with other drugs, which is also confirmed by the results of this meta-analysis [17]. In addition, esketamine can inhibit the production of pro-inflammatory factors and selectively block nociceptive signals from the reticular tract of the spinal cord, blocking pain transmission to the thalamus and cortical areas and producing analgesic effects [18]. It has also been reported that esketamine is able to agonize opioid receptors and produce analgesic effects, so esketamine should be effective in reducing the injection pain of propofol when it is injected before propofol, and the results of this meta-analysis verified this point [12]. Therefore, the use of esketamine significantly alleviates and compensates for the major side effects of propofol.

One of the non-negligible side effects of clinical induction doses of esketamine is the neuropsychiatric reaction during the awakening period, which is due to the ability of esketamine to activate prefrontal glutamate neurotransmission [19]. The use of subclinical doses definitely alleviates this side effect, and the results of the meta-analysis confirm that the combination of subclinical doses of esketamine and propofol does not increase the incidence of neuropsychiatric reactions compared to other matching regimens. In addition, esketamine increases cerebral blood flow and cerebral metabolic rate, and intracranial pressure increases with cerebral blood flow, while propofol has the effect of decreasing cerebral blood flow, cerebral oxygen metabolic rate and intracranial pressure, so propofol is able to reduce this side effect of esketamine [15]. In addition, there are some studies showing that esketamine has a rapid antidepressant effect, which may have a very positive meaning for some patients [20, 21]. Therefore, we can find that the use of propofol also greatly alleviates and compensates for the major side effects of esketamine.

Based on the results of the meta-analysis, we also found that the combination of propofol and subclinical doses of esketamine attenuated the incidence of intraoperative choking and intraoperative somatic movements, but the combination did not show differences in time to awakening, nausea and vomiting, and headache and dizziness compared with other pairing regimens. The combination of propofol and esketamine does not theoretically conflict with each other because they act on different receptor pathways, and their pharmacological properties suggest that they should compensate for each other’s deficiencies. The results of the meta-analysis confirmed that the overall incidence of adverse reactions was lower with subclinical doses of esketamine and propofol than with other combinations, and that no more serious side effects occurred.

In clinical practice, propofol is used in a wide range, and the dose has to be adjusted according to the patient’s specific situation, and the actual clinical dose range is about 1-2.5 mg/kg [22]. our specific dose of propofol use is oriented to the depth of patient sedation, so subclinical doses of esketamine and propofol combined, we cannot strictly limit the dose of propofol use, which is the reason why we did not use the dose of propofol as a variable for the screening of the literature when we performed the meta-analysis. We suggest that, in clinical practice, esketamine is injected first, followed by propofol. First, the injection of esketamine first can significantly reduce the injection pain of propofol, and second, because we mainly achieve our desired depth of sedation with propofol, and the use of esketamine is mainly to alleviate some side effects of propofol, we can limit the dose of esketamine, thus providing an effect that can alleviate the side effects of propofol without causing the side effects that esketamine itself. The optimal dose of subclinical esketamine is currently considered by most studies to be 0.3 mg/kg, which may require more randomized controlled trials to verify [23].

We found through our search that although there have been many randomized controlled trials that have confirmed that subclinical doses of esketamine and propofol combined significantly reduce the incidence of various adverse reactions compared with the combination of propofol and other drugs, no higher level of evidence has been found to validate this conclusion. Therefore, this meta-analysis is groundbreaking, although it still has many imperfections. We look forward to the emergence of more randomized controlled trials in the future to further update and improve the issues analyzed in this meta.

Our study is not without limitations. First, most of the studies we included were from China, which may lead to poor extrapolation of the findings and potentially large publication bias. Second, we included studies with a wide range of subjects’ ages and did not strictly distinguish between younger and older adults, which may have affected the results to some extent, so we excluded one study with all subjects in the older age group and found that the meta results for each outcome indicator containing this study did not reverse after excluding this study, suggesting that this study did not seriously affect the results of the meta. We would have liked to perform a subgroup analysis because the drugs used in the control group and propofol were different in each study, but we abandoned the subgroup analysis because of the small sample size of each subgroup.

## Conclusion

In non-intubated general anesthesia, the combination of subclinical doses of esketamine and propofol did reduce circulatory and respiratory depression, injection pain, and other adverse effects, while the incidence of esketamine’s own side effects such as neuropsychiatric reactions did not increase, and the combination of the two did not cause the occurrence of new and more serious adverse reactions, and the combination of the two was safe and effective.

## Data Availability

The datasets used and analysed during the current study are available from the corresponding author on reasonable request.
