# Correction: Analysis of the efficacy of subclinical doses of esketamine in combination with propofol in non-intubated general anesthesia procedures - a systematic review and meta-analysis

**DOI:** 10.1186/s12871-023-02326-3

**Published:** 2023-10-31

**Authors:** Haoming Chen, Xizhi Ding, Guilin Xiang, Liu Xu, Qian Liu, Qiang Fu, Peng Li

**Affiliations:** 1 Department of Anesthesiology, Affiliated Hospital of Southwest Medical University, Luzhou, China; 2 Department of Anesthesiology, Sichuan Provincial People’s Hospital, Sichuan Academy of Medical Sciences, University of Electronic Science and Technology of China, Chengdu, China; 3 Chengdu Medical College, Chengdu, China; 4 Wenjiang Hospital of Sichuan Provincial People’s Hospital, Chengdu, China; 5 Department of Anesthesiology, The Third People’s Hospital of Chengdu, Chengdu, China; 6 Department of Anesthesiology, The First People’s Hospital of Guangyuan, Guangyuan, China

**Correction: BMC Anesthesiol 23, 245 (2023)**

10.1186/s12871-023-02135-8

Following publication of the original article [1], the authors reported an error found in affiliation 1. The correct affiliations should be “Department of Anesthesiology, Affiliated Hospital of Southwest Medical University, Luzhou, China” instead of “Southwest Medical University, Luzhou, China.”.

The original article [1] has been updated.
